# Golf and upper limb injuries: a summary and review of the literature

**DOI:** 10.1186/1746-1340-13-7

**Published:** 2005-05-25

**Authors:** Andrew J McHardy, Henry P Pollard

**Affiliations:** 1 Macquarie Injury Management Group Macquarie University, Sydney 2109 Australia

**Keywords:** Golf, injury, shoulder, elbow, wrist, review

## Abstract

**Background:**

Golf is a popular past time that provides exercise with social interaction. However, as with all sports and activities, injury may occur. Many golf-related injuries occur in the upper limb, yet little research on the potential mechanisms of these injuries has been conducted.

**Objective:**

To review the current literature on golf-related upper limb injuries and report on potential causes of injury as it relates to the golf swing.

**Discussion:**

An overview of the golf swing is described in terms of its potential to cause the frequently noted injuries. Most injuries occur at impact when the golf club hits the ball. This paper concludes that more research into golf-related upper limb injuries is required to develop a thorough understanding of how injuries occur. Types of research include epidemiology studies, kinematic swing analysis and electromyographic studies of the upper limb during golf. By conducting such research, preventative measures maybe developed to reduce golf related injury.

## Introduction

Golf is a popular recreational activity that can be played by all ages, genders, and skill levels. Although seemingly uncommon, golf-related injuries do occur, with the three most common injury sites being the lower back, the elbow and the wrist. Together these three sites account for approximately 80% of all injuries sustained by golfers [1-4]. While a number of investigators have conducted research into back-related golfing injuries [5-8] and reviewed how these injuries were sustained [9,10], little research has been identified on how golfing injuries occur in the elbow and wrist [11,12]. The purpose of this paper is to review the function of the upper limb during the golf swing. Also presented is a review of golf-related injuries of the wrist, the elbow and the shoulder as they relate to the golf swing. Finally, there is a discussion on avenues for potential research to understand golf-related upper limb injuries.

## Methods

A search of the literature was conducted in the following databases: Medline, Cinahl and Mantis (1966 to present, 1982 to present and 1980 to present respectively). A search of the terms: golf and injury and shoulder or elbow or wrist revealed 45 papers. After setting criteria that required blinded peer-reviewed English language journals only, 42 papers were eventually selected. The literature was collated and sorted according to injury site and relevance. The reference lists of selected papers were examined to determine if any reference papers not found in the original search were relevant. The authors conducted an assessment of methodology and shortcomings of studies, with the findings presented in the discussion section.

### The golf swing

The golf swing is a dynamic movement with the potential to cause injury to the golfer. Various injuries occur in different sections of the swing and frequently involve soft tissue injuries [1-4]. An understanding of the mechanics of the golf swing will facilitate appropriate knowledge of the etiology of the injury, thereby improving management. This is particularly true of upper limb golf-related injuries as the arms go through a large range of motion (ROM) during the swing, while providing the link between the fast moving club and the power-generating torso.

**Figure 1:**
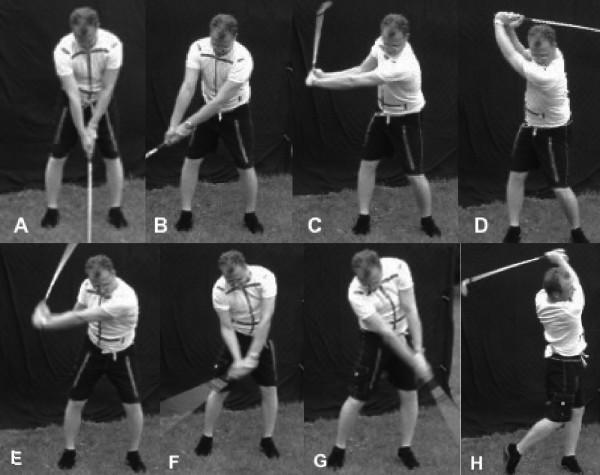
A-H. Phases of the golf swing. A. Address position, B. Early backswing, C. Late backswing, D. Top of swing, E. Downswing, F. Acceleration, G. Early follow-through, H. Late follow-through.

The golf swing can be defined as the process of swinging the club to hit the ball. Other than the address position (Figure 1A) it can be divided into seven parts: early backswing (Figure 1B), late backswing (Figure 1C), top of swing (Figure 1D), downswing (Figure 1E), acceleration (Figure 1F), early follow-through (Figure 1G), and late follow-through (Figure 1H).

The golf swing is also often divided into 5 phases: the backswing, the downswing, acceleration, early follow-through and late follow-through [9,13]. In the right-handed golfer, the backswing results in the club being moved away from the direction of intended ball flight and is characterised by a rotation of the shoulder girdle to the right. There is resulting right arm abduction, flexion and external rotation with corresponding left arm adduction, flexion and internal rotation. This takes the golf club in the desired direction. To achieve this movement, the right scapula retracts, while the left scapula protracts and this allows their movement around the trunk in a clockwise movement. The muscles that are predominantly active in this phase and produce these movements are upper and middle trapezius on the right, and the subscapularis and serratus anterior on the left [14-18].

At the top of the backswing, the wrists are in radial deviation, with the right wrist also displaying submaximal extension (Figure 1D).

The downswing phase starts from the top of the backswing and involves the club returning along a similar path to the backswing in preparation to hit the ball, and it involves rapid arm movement. The combined movement of left rotation of the shoulder girdle and scapular rotation, in an anti-clockwise direction around the trunk, is required during the downswing, resulting in increased activity in the left medial scapulae stabilisers/ retractors. To achieve right-sided internal shoulder rotation and flexion, the pectoralis major is very active, while the right upper serratus anterior contracts to assist scapular protraction [14-18].

As seen in Figure 1F, the wrists remain in a similar position to the top of the backswing, a position that is termed 'cocked'.

The acceleration phase of the golf swing is the continuation of the downswing to ball impact. The club head is accelerated to its peak velocity in this phase just prior to contact with the ball, making this the most active phase of the entire golf swing. Bilaterally, the pectoralis muscles are the most active muscles, being the major movers of the shoulder girdle. There is continuation of the right side activity seen during the early downswing, while the left pectoralis appears to maintain an eccentric contraction to control the left arm abduction and external rotation. The muscles involved in scapular movement are also active: the upper serratus on the right to protract the scapula and the levator scapulae on the left side to aid scapular tilting [14-18]. Just prior to impact there is a large increase in wrist flexor muscle activation; what has been termed the 'flexor burst' [11,19,20]. Part of this activity is to return the wrists back (thus club head back) to a position to hit the ball, the 'uncocking' of the wrists.

The early follow-through of the golf swing occurs after ball impact and is the phase where deceleration of trunk rotation occurs. There is a 'rolling' of the forearms at impact that is continued into the early follow-through. This results in left arm supination and right arm pronation followed by left arm external rotation and right arm internal rotation. Bilaterally, the pectoralis major muscles continue to be very active. The active muscles in the shoulder during this phase are the right subscapularis and the left infraspinatus to control the movement seen in the follow-through [14-18].

In the late follow-through, the muscle activity decreases as the golfer nears the end of the swing. The most active muscles in this phase are similar to the early follow-through, but with a lesser degree of activity. The only exception in the upper body is the right serratus anterior, which is more active in this phase as it aids in the protraction of the scapular around the trunk [14-18].

### Wrist/Hand injuries

The wrist is one of the most common sites of injury in golfers [3,4]. The wrist accounts for 13–20% of all injuries in amateurs and 20–27% of all injuries in professionals in golf injury epidemiology studies [1-4]. During the golf swing, the wrist is the anchor point between the club and the body. This results in the wrist displaying a large range of motion [19,20]. Wrist injuries commonly occur at the impact point of the golf swing and may result from hitting an object other than the ball. The injury is the result of the sudden change in load applied to the club, and subsequently the golfer, resulting in tissue disruption to the hands and wrist. This commonly occurs in amateurs due to hitting the ball 'fat' (i.e., hitting the ground before the ball). Professionals also sustain wrist injuries but these injuries usually occur in slightly different circumstances. The professional (or amateur) may hit an obscured rock whilst playing from 'the rough' (longer grass that borders the shorter grass of the fairway, the central area that is preferable to hit from). In many major tournaments, particularly "links" courses commonly seen in the United Kingdom, the rough tends to be thick. Whilst attempting to extricate the ball, the long strands of grass tend to wrap themselves around the hosel (that part of the club that joins the shaft to the club head) and shaft of the club. This results in a similar deceleration of the club head during the downswing as hitting the ground, which lends itself to injury. Injury may be either acute where enough force is produced to cause excessive soft tissue elongation in a single swing, or by way of repetitive microtrauma if repeated many times in a short timeframe. Injuries of this nature tend to occur at the hand and wrist but can also occur at the elbow. Muscular strains (particularly the flexor carpi ulnaris [FCU]) and ligamentous strains are common [21,22], but fractures of the hook of hamate may also occur due to this mechanism [23].

Overuse injuries to the wrist are also common and are due mainly to repetitive wrist movement during practice or from alteration to the swing that results in stress to unaccustomed areas. According to a study of the Spain National Insurance Scheme for sportsmen, 10% of golf injuries occur in the wrist. This is contrary to the statistics produced in golf epidemiology studies. A reason for this difference could be differing definitions of what an injury is in each study. The Spanish study found that overuse or sudden changes in swing were the common injury mechanisms, and the FCU was the most common site of injury [21].

Tendonopathy, or more specifically tendonosis has replaced tendonitis as the clinical descriptor of the overuse syndrome [24,25]. The primary reason for this change is due to the majority of overuse tendonopathies displaying collagen degeneration and fibre disorientation. However they do not display the presence of inflammatory cells [24], hence the "itis" is inaccurate. The injury mechanism is either a sudden increase in the volume of practice or alteration of the grip (causing increased loading on an unaccustomed part of the wrist), and then subsequent practice [26]. Onset of the pain is gradual. It tends to have a persistent nature until any aggravating factor(s) are modified or adequate repair (healing) time elapses [24-26].

The FCU of the right wrist in right-handed golfers is vulnerable to injury from microtrauma due to the large forces produced by the swing just prior to impact. This is particularly true when golfers take divots (hit the ground) [26]. As the club hits the ground, a sudden resistance occurs that loads the flexor tendon. If the forces are great enough microtrauma can occur, which combined with repetition through practice may lead to injury. Injury to the FCU results in pain at the proximal border of the trapezium and is increased with wrist flexion.

In the presence of a faulty swing style, the beginner is also susceptible to extensor carpi ulnaris (ECU) injury [26]. Commonly, the beginner 'casts' the club in the early downswing (the early uncocking of the wrist during the downswing and a source of lost power and control), which loads the ECU [26]. Beginners are often overenthusiastic in their practice in an endeavour to improve their game. This may result in repetitive loading, microtrauma and injury to the ECU. A sign of ECU injury includes ulnar wrist pain with tenderness of the dorsal base of the ulnar styloid where the ECU runs through the sixth dorsal compartment. There is often pain on resisted supination and on ulnar deviation in this instance.

An uncommon injury seen in golfers is a fracture to the hook of hamate. Hamate fractures may be acute in nature due to the impingement of the hamate between the hand and the butt end of the club, leading to a fracture in the leading hand (the left hamate in a right-handed golfer) [23]. The literature records acute hamate fractures in golfers as early as 1972 [23]. Stress fractures of the hamate may also occur due to a sudden change in grip positioning or strength with accompanying excessive practice [27]. The ulnar border of the wrist is the site of pain for hamate fractures, with hamate tenderness and positive percussion being an indication for imaging. Care must be taken, however, as x-rays may initially not reveal the fracture [28]. Bone scans or MR imaging will show the fracture.

Other unusual golf-related injuries to the wrist and surrounding structures have also been reported in the literature. These include a case of an amateur golfer with a compression neuropathy of the median nerve in the right palm due to mechanical compression of the median nerve in the right palm by the head of the first metacarpal bone of the left hand [29]. Extensor carpi ulnaris (ECU) tendon dislocation over the ulnar dorsal ridge of the ulnar head aggravated by excessive practice has also been reported [30]. This case was resolved by extensor retinaculum release and partial ulnar head resection after conservative therapy failed. The unusual "hypothenar hammer syndrome" has also been reported in a golfer due to the repetitive hitting of practice balls with a 'faulty' grip causing repeated pressure on the ulnar artery underlying the hypothenar eminence. This practice resulted in thrombus formation in the ulnar artery [31]. While unusual, putting grip alterations have resulted in pain to the volar radial wrist due to a flexor carpi radialis strain. It was reported that this was accentuated by palpation and that a return to the original grip with manual therapy resolved the condition [32].

### Elbow injuries

Elbow injuries are common in golfers, especially in amateurs and particularly in females. This is thought to be due to the increased carrying angle seen in the female population [3]. Elbow injuries account for 25–33% of all injuries in amateurs and 7–10% of all injuries in professionals. Ironically, lateral elbow injuries are more common, at a rate of 5:1 when compared to medial elbow injuries (including the so-called Golfer's elbow) [2].

Medial elbow injuries are thought to result from traction-based insults to the elbow, usually to the trailing arm (right elbow in the right-handed golfer). It is the wrist/hand flexors and forearm pronators that are injured at their insertion into the medial epicondyle. These injuries are usually of a traumatic nature and occur at the time of impact. The mechanism is a sudden deceleration of the club head, leading to an increased loading of the medial elbow. This can be due to hitting obscured rocks and tree roots, and in professionals trying to hit repeatedly out of long and thick rough. With amateurs, the hitting of a 'fat' shot is the more likely mechanism. Signs of medial epicondylitis (Golfer's elbow) include pain and tenderness to palpation of the medial epicondyle. Pain is often aggravated by resisted forearm flexion and forearm pronation. There may be trigger point referral along the radial border of the forearm into the dorsum of the hand.

Injury of the lateral aspect of the elbow, the insertion of the wrist/hand extensors into the lateral epicondyle, is more likely to be due to overuse [33]. Gripping the club too tightly during the swing (causing associated extensor eccentric contraction) or changes to the grip with subsequent practice (often fatigue-based) may result in changes in forearm musculature forces and are potentially a source of lateral epicondylitis. Signs of lateral epicondylitis include pain and tenderness to palpation of the lateral epicondyle. Pain is often aggravated by resisted forearm extension and on occasions gripping objects or shaking hands. There may be trigger point referral along the ulnar border of the forearm into the palmar aspect of the hand.

Excessive practice may also result in injury to the lateral elbow. The large increase in flexor activity just prior to impact, the 'flexor burst' [11] accompanied by the rapid wrist movement at the same time places a large stress on the elbow joint and may result in injury due to accumulating microscopic damage [34].

Even though the elbow is a common injury site in golfers, little research has been conducted in this area. Most of the elbow injury mechanisms and management plans are based on racquet sports related injuries. Research focusing on the mechanics of the elbow and related musculature would allow for the accurate aetiology of golf-related elbow injuries to be determined. Understanding how these injuries occur in golfers would ensure the development of appropriate management strategies targeting golf specific injury mechanisms.

### Shoulder injuries

Shoulder pain in golfers is a relatively common occurrence compared to other sites of the body, accounting for approximately 8–18% of all golf injuries [1-4].

The shoulder goes through a large ROM during the golf swing including a large degree of left shoulder horizontal adduction and right shoulder external rotation in the backswing. In the follow-through, there is a large degree of left shoulder external rotation and horizontal abduction and right shoulder horizontal adduction [35]. Consequently, excessive practice can produce problems of the shoulder due to overuse.

Injuries to the shoulder in golfers are mainly restricted to the lead shoulder, the left shoulder in right-handed golfers. Studies have found that shoulder pain may be localised to the acromioclavicular (AC) joint, with the potential for either osteoarthritis or distal clavicle osteolysis (which implies horizontal plane compression loading of the joint) [36]. A second study found that posterior instability and subacromial impingement were common findings in golfers with shoulder pain [37]. This pain and instability were reproduced at the top of the backswing (maximal horizontal adduction) [37]. Previously, Bell found that maximal forces about the AC joint occurred in horizontal abduction and adduction. Similar positions are attained by the arm at the top of the back swing (left arm horizontal adduction) and at the end of the follow-through (left arm horizontal abduction), which emphasizes the potential for injury to the AC joint by excessive practice of the golf swing [38].

The practitioner should ascertain the phase of the golf swing that produces the patients shoulder pain; this may facilitate the diagnosis [39]. Posterior shoulder pain in the left shoulder of a right-handed golfer at the top of the backswing should alert the clinician to tightness of the rotator cuff musculature, tightness of the posterior capsule, or posterior capsulitis [39]. Anterior joint line pain at the top of the backswing implies impingement of the humeral head and anterior labrum, while pain localised to the AC joint indicates possible degeneration or impingement of the AC joint [39].

The follow-through phase of the swing may produce posterior shoulder pain due to impingement of either the posterior labrum or the underside of the rotator cuff muscles [39]. Shoulder pain that is generalised and occurs throughout the swing may be due to scapular lag, which alters the mechanics of the shoulder during the swing [39].

A study of golfers who underwent shoulder arthroplasty and were able to return to golf, found that the right shoulder was operated on more frequently (14 out of 26). However, this study made no mention of the cause of the patients shoulder pain. The study also asked a group of surgeons about their opinion of the patient returning to golf after arthroplasty. Out of 44 respondents, 91% encouraged a return to play. This survey showed that shoulder arthroplasty does not necessarily prohibit a return to golf [40].

It is noteworthy that a lack of trunk rotation may require the much smaller shoulder rotators to become excessively active to maintain the momentum of the golf swing. Such a scenario would likely result in the shoulder dysfunction frequently noted in golfers, particularly instability in professionals. It is also worthy to note that those with back problems may potentially induce a shoulder problem in their attempt to reduce the loads on a painful back. Baulbian noted similar observations in his research on a modified golf swing where the back swing is shortened. This research reported that the forces generated in the lower back were reduced by this swing, but the forces generated in the shoulder were greater [41]. This results in the potential for this swing to produce shoulder injury that maybe the result of impingement, instability or rotator cuff tendonopathy. Pain location and shoulder orthopaedic testing helps to differentiate between each clinical entity, though MRI is required to provide a definitive diagnosis.

## Discussion

On examining the literature on golf injuries to the upper limb, it is apparent that the majority of papers are case report-based. A case study reports on an individual patient's outcomes and as a result there are inherent limitations such as a lack of control and an inability to generalize findings to the whole population. This type of study, however, provides a platform for the establishment of a testable hypothesis to be made with further research [42]. The studies on golf injury epidemiology allow for a comparison of injury frequency to specific injury sites and also between different groups of golfers (based on gender, skill and age). Most of these studies are retrospective in nature. These types of studies allows for a great deal of data to be gathered, but are susceptible to recall bias. Recall bias occurs when what is thought to have occurred in the past is different to what truly occurred. The use of prospective studies would dramatically reduce recall bias.

How the data are collected influences the accuracy of the data set. Response rates influence how well the results collected can be extrapolated to the population in question. The higher the response rate, the more likely the data are applicable to the whole population in question. Response rates were generally poor ranging from 20.6% to 43%. However, if the sample size is large enough, such data may still be helpful when comparing sites and rates of injury.

An anonymous survey sent in the mail is more likely to be accurate, when compared to a personal interview, particularly with sensitive questions. The majority of the epidemiology studies cited use an anonymous mailed survey that was sent to a group of golfers.

It is apparent that little direct research has been conducted into golf-related upper limb injuries. Much of what if known about injuries relating to the upper limb comes from studies of racquet sports, particularly tennis. While a number of studies have analysed muscle activity in the shoulder musculature during the golf swing, the studies analysed the swing of professional/elite golfers. In many cases, this data may not be applicable to the 'average' golfer (e.g. handicap of 18) due to a difference in the golf swing. To overcome this, research on the swing of the 'average' golfer concentrating on what occurs at the shoulder is needed. This type of study should also look at different swing types: the modern swing, the classic swing and the more recent hybrid swing. Many injuries in golf relate to the wrist and elbow and occur at impact during the golf swing. Research into the forces that occur in the 'perfect' swing and also what occurs in different types of swings/incidents such as miss hits and hitting the ground could provide information on why such injuries occur. Data collected in the research mentioned above may inform injury management (including conditioning / rehabilitation programs) and also potentially prevent upper limb injuries during golf.

## Conclusion

The golf swing is a complex body movement involving a large ROM of the upper limb that acts as a link between the golf club and the body. Injuries to the upper limb account for the majority of golf-related injuries recorded. Many injuries occur as the club impacts the ball and are muscle-related. An understanding of how the body moves and the muscle activity achieved during the golf swing helps the health practitioner to understand why these injuries occur. Further study into the different types of golf swing and the different skill levels of golfers is required to fully understand the upper limb function in the golf swing. Such understanding may enable the development of management and prevention programs to reduce the upper limb injuries caused by golf.

## Authors' contributions

AJM: Conception and design, search data, paper collection, drafting manuscript, final approval.

HPP: Conception and design, search data, critical review of manuscript, final approval.

## References

[B1] Gosheger G, Liem D, Ludwig K, Greshake O, Winkelmann W (2003). Injuries and overuse syndromes in golf. Am J Sp Med.

[B2] McCarroll, Retting AC, Shelbourne KD (1990). Injuries in the amateur golfer. Phys Sports Med.

[B3] Batt ME (1992). A survey of golf injuries in amateur golfers. Br J Sports Med.

[B4] McCarroll JR, Gioe TJ (1982). Professional golfers and the price they pay. Phys Sports Med.

[B5] Sugaya H, Tschiya A, Moriya H, Morgan DA, Banks SA, Farrally MR, Cochran AJ (1998). Low-Back Injury in Elite and Professional Golfers An Epidemiologic and Radiographic Study. Proceedings of the World Scientific Congress of Golf Science & Golf Ill: 20–24 July 1998; St Andrews.

[B6] Burdorf A, Van Der Steenhoven GA, Tromp-Klaren EG (1996). A one-year prospective study on back pain among novice golfers. Am J Sports Med.

[B7] Leigh RJ, Young DB, Farrally MR, Cochran AJ (1998). Back pain among junior golfers. Proceedings of the World Scientific Congress of Golf Science & Golf Ill: 20–24 July 1998; St Andrews.

[B8] Hosea TM, Gatt CJ, Galli NA, Cochran AJ, E, FN (1990). Biomechanical analysis of the golfer's back. Proceedings of the World Scientific Congress of Golf Science & Golf I: 20–24 July 1990; St Andrews.

[B9] McHardy A, Pollard H (2005). Lower back pain in golfers: A review. J Chiro Med.

[B10] Hosea TM (1996). Back pain in golf. Cl Sports Med.

[B11] Glazebrook MA, Curwin S, Islam MN, Kozey J, Stanish WD (1994). Medial epicondylitis. An electromyographic analysis and an investigation of intervention strategies. Am J Sports Med.

[B12] Loftice J, Fleisig GS, Zheng N, Andrews JR (2004). Biomechanics of the elbow in sports. Clin Sports Med.

[B13] McHardy A, Pollard H, Luo K (2005). Golf injuries: A review. Sports Med.

[B14] Kao JT, Pink M, Jobe FW, Perry J (1995). Electromyographic analysis of the scapular muscles during a golf swing. Am J Sports Med.

[B15] Pink M, Jobe FW, Perry J (1990). Electromyographic analysis of the shoulder during the golf swing. Am J Sports Med.

[B16] Jobe FW, Perry J, Pink M (1989). Electromyographic shoulder activity in men and women professional golfers. Am J Sports Med.

[B17] Moynes DR, Perry J, Antonelli DJ, Jobe FW (1986). Electromyography and motion analysis of the upper extremity in sports. Phys Ther.

[B18] Jobe FW, Moynes DR, Antonelli DJ (1986). Rotator cuff function during a golf swing. Am J Sports Med.

[B19] Cahalan TD, Cooney WP, Tamai K, Chao EY (1987). Biomechanics of the golf swing as related to club handle design. Biomechanics in sport.

[B20] Cahalan TD, Cooney WP, Tamai K, Chao EY (1991). Biomechanics of the golf swing in players with pathologic conditions of the forearm, wrist, and hand. Am J Sports Med.

[B21] Anonymous (1977). Golfers' wrist. Br Med J.

[B22] Skolnick AA (1998). 'Golfer's wrist' can be a tough break to diagnose. JAMA.

[B23] Torisu T (1972). Fracture of the hook of the hamate by a golfswing. Clin Orthop.

[B24] Maffulli N, Wong J, Almekinders LC (2003). Types and epidemiology of tendinopathy. Clin Sports Med.

[B25] Clancy WG, Hagan SV (1996). Tendinitis in golf. Clin Sports Med.

[B26] Murray PM, Cooney WP (1996). Golf-induced injuries of the wrist. Clin Sports Med.

[B27] Guha AR, Marynissen H (2002). Stress fracture of the hook of the hamate. Br J Sports Med.

[B28] Walsh JJ, Bishop AT (2000). Diagnosis and management of hamate hook fractures. Hand Clin.

[B29] Hsu WC, Chen WH, Oware A, Chiu HC (2002). Unusual entrapment neuropathy in a golf player. Neurology.

[B30] Oka Y, Handa A (2001). Recurrent dislocation of the ECU tendon in a golf player: release of the extensor retinaculum and partial resection of the ulno-dorsal ridge of the ulnar head. Hand Surg.

[B31] Mueller LP, Mueller LA, Degreif J (2000). Hypothenar hammer syndrome in a golf player: A case report. Am J Sports Med.

[B32] McHardy A, Pollard H (2004). Unusual cause of wrist pain in a golfer. Br J Sports Med.

[B33] Stockard AR (2001). Elbow injuries in golf. J Am Osteopath Assoc.

[B34] Stover CN, Wiren G, Topaz SR (1976). Modern golf swing and stress syndromes. Phys Sportsmed.

[B35] Mitchell K, Banks S, Morgan D, Sugaya H (2003). Shoulder motions during the golf swing in male amateur golfers. J Orthop Sports Phys Ther.

[B36] Mallon WJ, Colosimo AJ (1995). Acromioclavicular joint injury in competitive golfers. J South Orthop Assoc.

[B37] Hovis WD, Dean MT, Mallon WJ, Hawkins RJ (2002). Posterior instability of the shoulder with secondary impingement in elite golfers. Am J Sports Med.

[B38] Bell R, Acus R, Noe D (1993). A study of acromioclavicular forces. J Sh Elbow Surg.

[B39] Jobe FW, Pink MM (1996). Shoulder pain in golf. Clin Sports Med.

[B40] Jensen KL, Rockwood CA (1998). Shoulder arthroplasty in recreational golfers. J Shoulder Elbow Surg.

[B41] Bulbulian R, Ball KA, Seaman DR (2001). The short golf back swing : effects on performance and spinal health implications. J Manipulative Physiol Ther.

[B42] Portney LG, Watkins MP (2000). Foundations of clinical research: Applications to practice.

